# ggstThe role of tendon microcirculation in Achilles and patellar tendinopathy

**DOI:** 10.1186/1749-799X-3-18

**Published:** 2008-04-30

**Authors:** Karsten Knobloch

**Affiliations:** 1 Plastic, Hand and reconstructive surgery, Hannover Medical School, Germany

## Abstract

Tendinopathy is of distinct interest as it describes a painful tendon disease with local tenderness, swelling and pain associated with sonographic features such as hypoechogenic texture and diameter enlargement. Recent research elucidated microcirculatory changes in tendinopathy using laser Doppler flowmetry and spectrophotometry such as at the Achilles tendon, the patellar tendon as well as at the elbow and the wrist level. Tendon capillary blood flow is increased at the point of pain. Tendon oxygen saturation as well as tendon postcapillary venous filling pressures, determined non-invasively using combined Laser Doppler flowmetry and spectrophotometry, can quantify, in real-time, how tendon microcirculation changes over with pathology or in response to a given therapy. Tendon oxygen saturation can be increased by repetitive, intermittent short-term ice applications in Achilles tendons; this corresponds to 'ischemic preconditioning', a method used to train tissue to sustain ischemic damage. On the other hand, decreasing tendon oxygenation may reflect local acidosis and deteriorating tendon metabolism. Painful eccentric training, a common therapy for Achilles, patellar, supraspinatus and wrist tendinopathy decreases abnormal capillary tendon flow without compromising local tendon oxygenation. Combining an Achilles pneumatic wrap with eccentric training changes tendon microcirculation in a different way than does eccentric training alone; both approaches reduce pain in Achilles tendinopathy. The microcirculatory effects of measures such as extracorporeal shock wave therapy as well as topical nitroglycerine application are to be studied in tendinopathy as well as the critical question of dosage and maintenance. Interestingly it seems that injection therapy using color Doppler for targeting the area of neovascularisation yields to good clinical results with polidocanol sclerosing therapy, but also with a combination of epinephrine and lidocaine.

## Introduction

This review focuses merely on the microcirculatory changes encountered in Achilles and patellar tendinopathy and its potential modification by different current treatment options. During the last years there has been tremendous research in this area. Approaches involved the term tendinosis which was defined from histopathologic findings involving widening of the tendon, disturbed collagen distribution, neovascularisation and increased cellularity [1,2]. The severity of these tendon changes encountered in tendinosis was quantified [3], and the importance of the ongoing process and cause of increased cell proliferation was demonstrated [4]. Based on these reports neovascularisation was 1 out of 4 criteria's of tendinosis, which I will refer to throughout this review.

### Neovascularisation in tendinopathy

Neovascularisation is one feature of tendinopathy among others at various anatomic sites, such as the Achilles tendon, the patella tendon, tendinopathy of the wrist as well as in tennis elbow. Contemporary ultrasound studies using colour and/or power Doppler ultrasound identified neovascularisation among patients suffering Achilles tendinopathy [5-7] as well as in histological specimens from Achilles tendon ruptures [8] (table 1).

**Table 1:** Distribution of Tendon Pathologic Scores in control and ruptured Achilles tendons

Variable	Control tendon (N = 46)				Ruptured tendon (N = 38)			
		
	0	1	2	3	0	1	2	3
Fiber structure	19	19	4	4	0	1	11	26
Fiber arrangement	19	19	4	4	0	1	11	26
Rounding of the nuclei	19	15	9	3	0	0	4	34
Regional variations in cellularity	23	12	8	3	0	0	9	29
***Increased vascularity***	***26***	***10***	***9***	***1***	***0***	***0***	***6***	***32***
Decreased collagen stainability	15	20	10	1	0	2	12	24
Hyalinization	19	21	5	1	0	5	25	8
GAG content	22	15	8	1	0	7	26	5

^a ^The worst scoring result was used in each situation.

Neovascularisation was also reported in ultrasound of patellar tendinopathy with the vessels typically arising from the Hoffa fat pad [9,10]. The same phenomenon has been described for lateral elbow tendinopathy [11], flexor carpi ulnaris tendinopathy of the wrist [12], posterior tibial tendon insufficiency [13], and in supraspinatus tendon overuse [14] determined by colour and/or power Doppler ultrasound techniques. Currently, there is reasonable published evidence that the neovessels are at least part of the pathophysiological process in tendinopathy of the Achilles tendon in its mid-portion area, at the patella tendon and in tendinopathies of the upper extremity such as in tennis elbow or in tendinopathies at the wrist level.

The diagnosis of tendinopathy of the main body of the Achilles tendon is made if patients have Achilles tendon pain at rest or at exercise in the main body of the Achilles tendon, 2–6 cm proximal to the insertion, associated with tenderness and swelling. In contrast, insertional tendinopathy of the Achilles tendon might involve various distinct clinical entities besides mere insertional tendon problems associated with neovascularisation. This distinct entity such as Haglund's exostosis or bursitis subachillae does not necessarily involve neovascularisation. Therefore, all insertional Achilles tendon problems reported in this review are tendon problems with neovascularisation arising from tiny vessels from the ventral aspect of the Achilles tendon in the Karger triangle with increased capillary blood flow.

The importance of structures close to the Achilles tendon and the "communication" in between and the role of the skin barrier, subcutis, as well as the paratenon is importance in this regard [15]. However, currently one has to be aware that the cells and biology which controls these extra and intra tendinous processes are only poorly understood. We do not even know what type of cells we find in the diseased tendons or how they work, and several up and down regulating factors, extrinsic and extrinsic factors may be involved.

### What drives the phenomenon of neovascularisation?

I use the term 'neovascularisation' as a descriptive term for the appearance of abnormal vessels [16] and 'angiogenesis' for the process by which this occurs. Angiogenesis is known to be controlled by several stimulatory and inhibitory proteins [17-19] (table 2). Inhibition of angiogenesis is necessary for the development and maintenance of hypo- or avascular tissues. This might be caused either by production of an inhibitory factor or by a reduction of the angiogenesis factor.

**Table 2:** Angiogenesis inhibitors and stimulators

**Angiogenesis inhibitors**	**Angiogenesis stimulators**
Chondromodulin-1	Metalloproteinase-9
Thrombospondin-1	Metalloproteinase-14
Thrombospondin.2	MT1-Metalloproteinase
Tissue inhibitor of metalloproteinases-1	Vascular endothelial growth factor-A
Tissue inhibitor of metalloproteinases -2	
Tissue inhibitor of metalloproteinases -3	

Endostatin	

The angiogenesis factor (vascular endothelial growth factor (VEGF) is expressed in fetal but not in adult tendons [20,21]. In adult tendons, the anti-angiogenesis factor endostatin is expressed [22] – especially in the gliding area of gliding tendons. Endostatin is a 20 kDa proteolytic fragment of collagen type XVIII with strong anti-angiogenic potency [23,24]. Endostatin inhibits proliferation, migration and apoptosis of endothelial cells. Endostatin also interacts with VEGF signal transduction by reducing VEGF-induced kinase (Erk1/2) phosphorylation [25]. Therefore, a complex balance between pro- and antiangiogenesis factors are involved in neovascularisation and this is reviewed by Pufe [26].

### Close relation between nerves and vessels

Mechanoreceptors and nerve-related components such as glutamate NMDA receptors are present in association with blood vessels in tendinopathic tendons [27,28]. In tennis elbow, substance P and its receptor (neurokinin-1-receptor) could be detected using immunostaining as well as interleukin-1 alpha and TGF beta 1 positive cells in small vessels [29]. Recently, the Umea research group described the distribution of general (PGP 9.5) and sensory (substance P/CGRP) innervations in the human patellar tendon [30]. They proposed that there was nerve-mediated regulation of the blood vessels supplying the tendon, at the level where they course in the loose paratendinous connective tissue. The same authors also demonstrated an up-regulation of the cholinergic system as well as the presence of autocrin/paracrine effects in patellar tendinopathy [31].

Recent publications suggest that the vascular in-growth in tendinopathy, in other words the neovascularisation, is accompanied with a nerval in-growth facilitating pain transmission in Achilles tendionpathy [32] and patella tendinopathy [33]. In other words we encounter a neuro-vascular inflammatory reaction in tendinopathy. Currently, based on the published reports, we cannot determine whether the vascularisation or the neurogenic component or both are the predominant factor in tendinopathy. One could speculate that with a resolution of the neovascularisation by a given treatment option such as eccentric training or sclerosing therapy, which I refer to later, the closely associated nerve endings will be disturbed or even destroyed due to a lack of perfusion by their nutrient neovessels. However, currently this is mere speculation. Alfredson speculated that eccentric training might traction the area of neovessels and be responsible for the good clinical results [34] but this hypothesis remains untested based on the current published reports.

### Diagnostic tools for microcirculatory assessment

Conventional ultrasound for tendon assessment in tendinopathy reveals hypoechogenic texture within an enlarged tendon especially in the anterior-posterior diameter. Power Doppler technology is capable in identifying neovascularisation in tendinopathic tendons because it allows visualisation of low-flow vessels by far more accurate than conventional colour Doppler ultrasound. In the Achilles tendon, these neovessels typically arise from the ventral and paratendinous portion leading into the Achilles tendon body. In patella tendinopathy these neovessels often arise inferior to the patella from the Hoffa fat pad entering the patella tendon in a 60°–90° angle. Magnetic resonance tomography determines tendon signal changes as well as paratendinous fluid with the signal intensity being the important factor in current tendinopathy MRI. Intratendinous pattern changes may be also depicted using MRI. Furthermore, volume calculations can be done using MRI, as demonstrated for the Achilles tendon by Shalabi [35].

Among 33 patients with chronic Achilles tendinosis (mean age 52 yrs) they found that a computerized 3-D seed growing technique demonstrates an overall excellent reliability to monitor and evaluate the volume of the Achilles tendon and the mean intratendinous signal. Furthermore, the same authors reported that both, eccentric and concentric loading of the Achilles tendon resulted in an immediately increased tendon volume and intratendinous signal in 22 patients with chronic Achilles tendinopathy [36]. The eccentric training regimen was performed with 3 sets of 15 repetitions of heavy-loaded eccentric training with an immediate MRI following this exercise within 30 minutes showing the above mentioned changes. However, one has to mention that acute tendon effects even within 30 minutes might not illustrate acute changes immediately after the exercise. Long term eccentric training decreases the Achilles tendon volume by 14% and the signal intensity in T1-weighted MRI scans from 6.6 ± 3.1 cm^3 ^to 5.8 ± 2.3 cm^3 ^(p < 0.05).

However, neither conventional ultrasound nor MRI is currently used for microcirculatory monitoring. Power Doppler is of qualitative use with visualisation of the course of the neovascularisation, but no quantitative data are derived by Power Doppler only in its current routine application.

### Microcirculation monitoring

**Figure 1:**
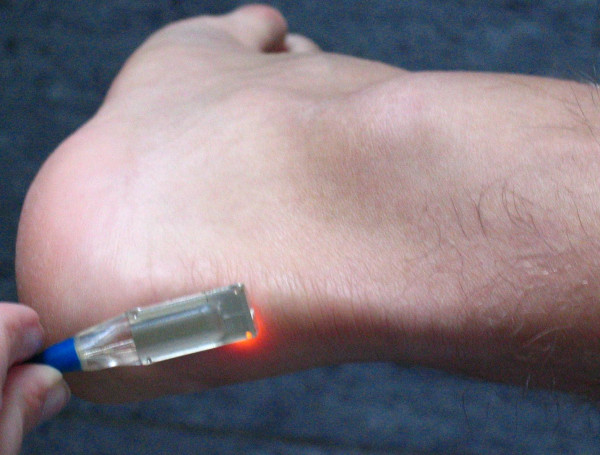
Oxygen-to-see probe, a combined laser Doppler and spectrophotometry system to determine Achilles microcirculation non-invasively.

**Figure 2:**
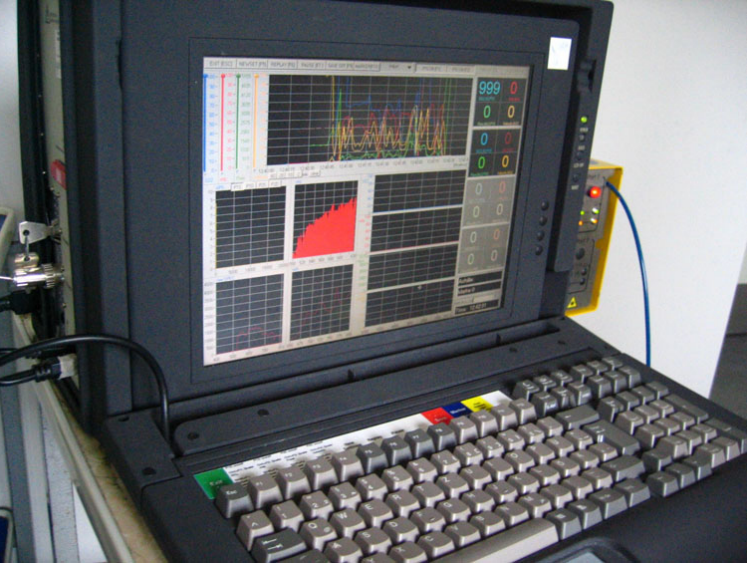
Oxygen-to-see system combining Laser Doppler flowmetry and spectrophotometry non-invasively to determine tendon capillary blood flow, tendon oxygen saturation, and tendon postcapillary venous filling pressures.

Real-time microcirculation assessment is possible using a combined non-invasive Laser-Doppler and spectrophotometry system, the Oxygen-to-see System (LEA Medizintechnik, Giessen, Germany, figures 1, 2). Three distinct parameters of microcirculation can be determined using the Oxygen-to-see system [37,38] (table 3):

**Table 3:** Overview regarding three microcirculatory changes and its physiological effect on the tendon.

**Microcirculatory change**	**Physiological effect on the tendon**
Capillary tendon flow↑	Potential harmful, increases pain by aggravation of neovascularisation
Capillary tendon flow↓	Beneficial, decreases pain by reducing neovascularisation, might harm the tendon at very low levels (threshold yet undetermined), achieved by cryotherapy and compression as well as eccentric training only
Tendon oxygenation↑	Beneficial, tendon oxygenation is increased, the resistance against ischemia is increased, hyperaemia is beneficial, achieved by combined cryotherapy and compression as well as eccentric training and Achilles wrap
Tendon oxygenation↓	Harmful, limits tendon oxygenation, increases lactate levels with acidosis, following ischemia
Postcapillary venous filling pressure↑	Harmful, increased pressure decreases clearance of local metabolic end products, consecutive increase in capillary flow following venous congestion, facilitating of infections and wound problems due to local stasis in venous congestion, increased in thrombosis and postthrombotic state
Postcapillary venous filling pressure↓	Beneficial, since clearance of metabolic end products is facilitated, achieved by cryotherapy and compression as well as by eccentric training and Achilles wrap

• Capillary flow

• Tissue oxygen saturation

• Postcapillary venous filling pressure

However, one has to consider that these three microcirculatory do not necessarily display the complete microcirculatory environment, since vascular factors such as clotting, adhesion, thrombus formation and several others are not addressed by the aforementioned mere non-invasive technique.

### Capillary blood flow

Laser-Doppler flowmetry has been introduced for determination of capillary flow in various disease states. Stern has applied the Doppler effect to study in the microcirculation as early as 1975 in his Nature paper [39]. Validation work has been done extensively in the following, stating that the Laser Doppler method is a promising tool for rapid monitoring of dynamic changes in tissue perfusion" [40].

Piloting the laser Doppler application to the tendon scientific area, Astrom and Svensson from the Malmo General hospital in Sweden studied the Laser Doppler flowmetry to the Achilles tendon surface of ten mature albino rats [41]. Clamping the femoral artery resulted in a 60% reduction of tendon blood flow and consecutive hyperaemia following clamp release in reperfusion. In circulatory arrest, no tendon flow was determined in this pilot study of Laser Doppler application in the tendon area.

Three years later, in 1994, Astrom and Westlin [42] reported about their initial experience at rest, during vascular occlusion, and during passive stretch and isometric contraction of the triceps surae among 40 healthy volunteers. They used an invasive needle probe, which was placed 5 mm above the distal insertion of the Achilles tendon, at the midportion and the musculotendineus junction of both legs, one of the first studies ever to differentiate between insertional and mid-portion locations. Astrom reported a significantly lower tendon blood flow at the insertion, but otherwise even vascular distribution. Vascular occlusion reduced all Achilles tendon blood flow values. Interestingly, passive stretch and isometric contraction induced a progressive decline in capillary tendon blood flow determined by laser Doppler flowmetry in this initial study. Hyperaemia was often noted by Astrom following contraction with higher tendon blood flow among women and a decreasing blood flow with increasing age. However, one has to bear in mind that only healthy volunteers participated in this study.

**Figure 3:**
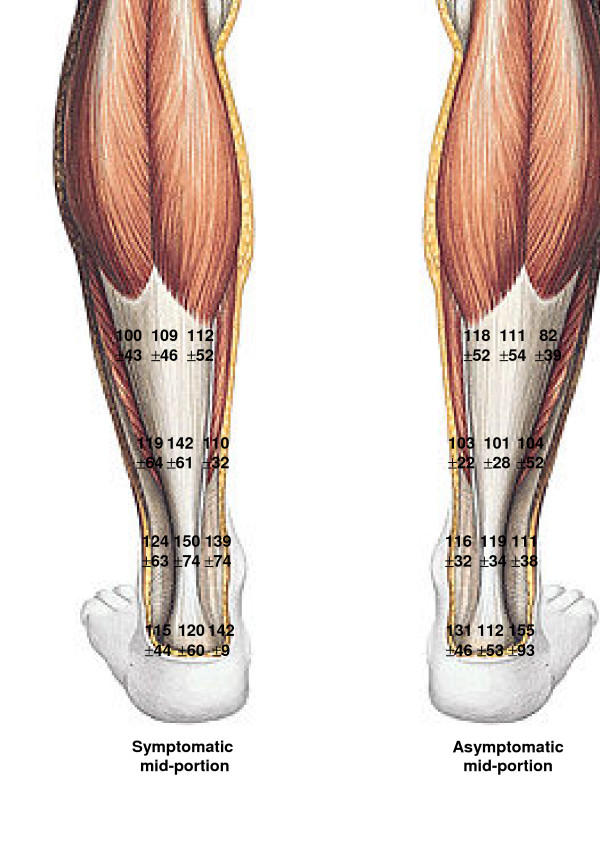
Capillary tendon blood flow in mid-portion symptomatic Achilles tendinopathy (left tendon) vs. the corresponding asymptomatic contralateral Achilles tendon in 50 patients with Achilles mid-portion tendinopathy.

**Figure 4:**
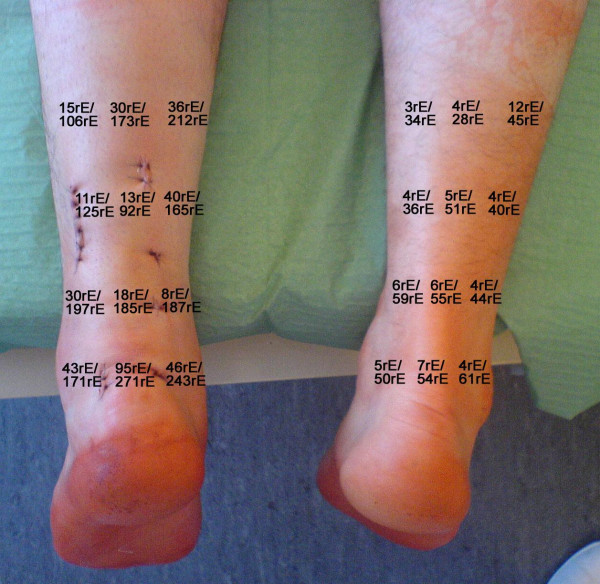
Superficial (upper numbers [rE as arbitrary unit]) and deep (lower numbers) capillary flow at the second postoperative day following minimal invasive percutaneous Achilles tendon repair at the left leg.

The reduced capillary tendon blood flow with increasing age is of special interest, since a decreased capillary tendon blood flow with increasing age might imply a consecutive malperfusion with age, thus leading to tendinopathy and finally to tendon rupture. On the other hand, as will be demonstrated in the following, we found that in symptomatic tendinopathy neovascularisation is associated with a significantly increased capillary blood flow in the Achilles tendon at the point of pain [43] (figure 3). Tendon repair, such as minimal-invasive percutaneous in complete Achilles tendon rupture, changes Achilles microcirculation in a time-dependent manner (figure 4).

The distribution of tendon capillary blood flow was performed in an Achilles tendon mapping technique, evaluating four tendinous and eight corresponding paratendinous location throughout the Achilles tendon. In contrast to histological data from staining suggesting that within the mid-portion part of the Achilles tendon the perfusion is limited [44,45], which favours Achilles tendon ruptures in the mid-portion area due to its relative malperfusion. This was stated by the anatomical studies by Lang and supported by a plastination study from Heidelberg, Germany [46]. They studied the vascular anatomy of eight human specimens suing a plastination perfusion method through the femoral artery. They identified the well-vascularized paratendon with a large number of intra- and extratendinous anastomosis. Microcirculatory effects on the capillary flow have been described in cardiac surgery, where retrosternal capillary flow is reduced by 50% following harvesting of the internal thoracic artery for coronary revascularisation [47]. Recently we could demonstrate the successful combined sclerosing therapy with polidocanol followed by a 12-week eccentric training in a tennis player suffering tremendous pain due to flexor carpi ulnaris tendinopathy [12].

Considering tendon oxygen saturation and postcapillary venous filling pressures besides capillary blood flow one has to acknowledge that each microcirculatory parameter is independent from each other. However, pathophysiological relations are evident such as in the case of ischemia, where a decrease of capillary blood flow due to arterial vessel obstruction is followed by a decrease of tissue oxygenation [48]. A venous stasis such as a venous thrombosis increases postcapillary venous filling pressures with consecutive decrease of oxygen saturation due to venous congestion and subsequent decreased capillary inflow in the further course.

### Tissue oxygen saturation

Tissue oxygen saturation is attributed as the local oxygen content of the focussed tissue. Tissue oxygen saturation has been determined using different probes and techniques. In 1987, Stone and coworkers from the Brigham and Women's Hospital in Boston, MA determined the tendon and ligament oxygenation using invasive polarographic oxygen sensors [49]. First, they started with tendon oxygen saturation determination in the Achilles tendon of the sheep, where they found a 100% (13 of 13 events) response rate to changes of blood flow with consecutive oxygen saturation reduction. Second, they moved to the human anterior cruciate ligament, where they placed the invasive probe in five human knees during routing total knee replacement. In 83% of the cases (5 of 6 events) the oxygen saturation response was appropriate to the tourniquet placement.

**Figure 5:**
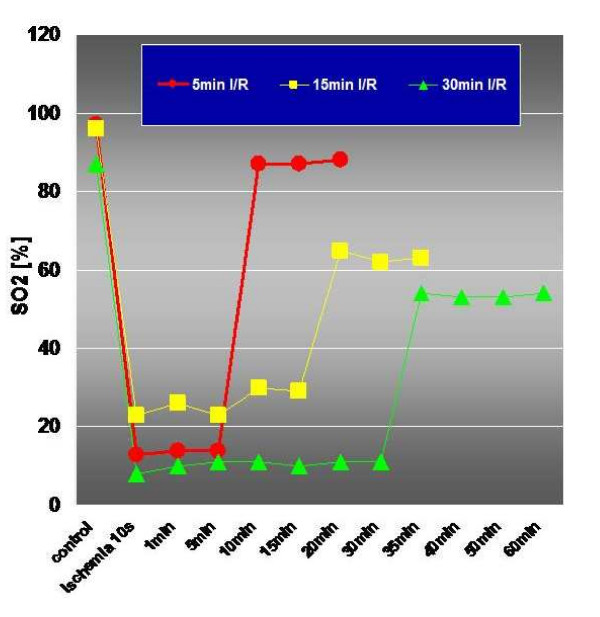
Myocardial oxygen saturation (SO2%) following 5 min (red), 15 min (yellow) and 30 min (green) of ischemia following clamping of the left descending coronary artery and reperfusion with decreased baseline myocardial oxygen saturation after 15 and 30 min of ischemia indicating an ischemia-induced damage to the myocardium (Knobloch K, unpublished data).

**Figure 6:**
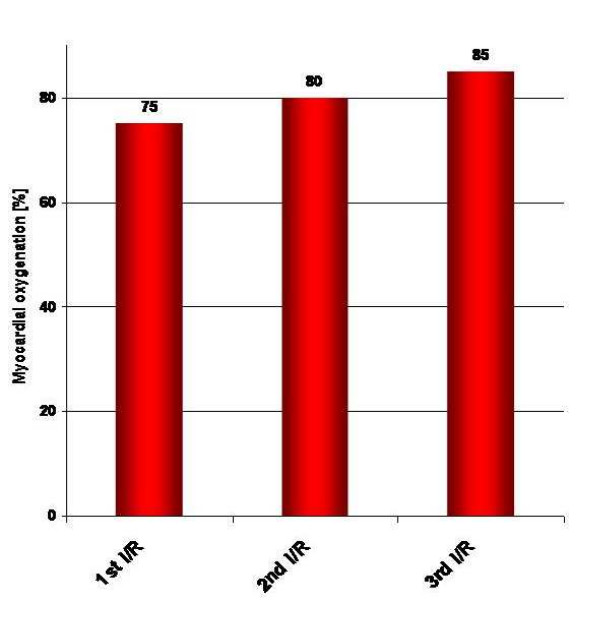
Myocardial oxygenation following preconditioning with 2 min of repetitive ischemia/reperfusion (I/R) following clamping of the left descending coronary artery in humans (Knobloch K, unpublished data)

Non-invasive tissue oxygen saturation is determined by spectrophotometry in the Oxygen-to-see System. Ischemia decreases oxygen saturation dramatically (figure 5). Repetitive ischemia and reperfusion, which is called preconditioning is capable in increasing tissue oxygenation (figure 6). Tendon oxygenation therefore is a marker for local oxygen content. A decrease of tendon oxygenation is potential harmful, since this is a sign for local acidosis. High intratendinous lactate levels have been reported in painful chronic Achilles tendinopathy by Alfredson^17^. Normal prostaglandin E2 levels have been identified by in vivo microdialysis as well] questioning the tons of non-steroidal anti-inflammatory drugs (NSAIDs) prescribed for this condition [50]. As aforementioned one has to acknowledge that tendon oxygen saturation may change independent of capillary blood flow as vice versa. Recently, an increase of Achilles tendon saturation has been reported after repetitive contractions among twelve men [51].

### Postcapillary venous filling pressures (rHb)

Venous congestion causes venous stasis, which is part of inflammation. Capillary venous stasis deteriorates local capillary clearance of local metabolic end products. On the other hand, decreased postcapillary venous filling pressures are beneficial, since local clearance is facilitated. In disease states, increased postcapillary venous filling pressures have been encountered in the retrosternal region following removal of the internal thoracic artery and vein for coronary revascularisation [48]. Decreased postcapillary venous filling pressures of the mid-portion Achilles tendon are encountered using simultaneous cryotherapy and compression over 10 minutes [52]. Achilles tendon postcapillary venous filling pressures were significantly reduced following a 12 week eccentric training at the Achilles tendon insertion (51 ± 16 vs.41 ± 19, p = 0.001) and the distal mid-portion (36 ± 13vs.32 ± 12, p = 0.037) at 2 mm and at the insertion of the Achilles tendon at 8 mm (63 ± 19vs.51 ± 13, p = 0.0001) [53].

#### Gender and Achilles tendon microcirculation

Based on the higher ligament injury rate among females vs. males such as for the anterior cruciate ligament injury we thought to evaluate the effect of gender on tendons. We found that symptomatic female patients suffering mid-portion Achilles tendinopathy have similarly elevated tendon capillary blood flow compared with symptomatic male patients suffering Achilles tendinopathy, but superior tendon and paratendon oxygen saturations and reduced postcapillary venous filling pressures indicate better tendon and paratendon Achilles tendon microcirculation in women [54]. Therefore, symptomatic females do not have worse, but equal or even superior Achillles tendon microcirculation compared to symptomatic males.

#### Tendinopathy treatment based on microcirculatory changes

##### Eccentric training

**Figure 7:**
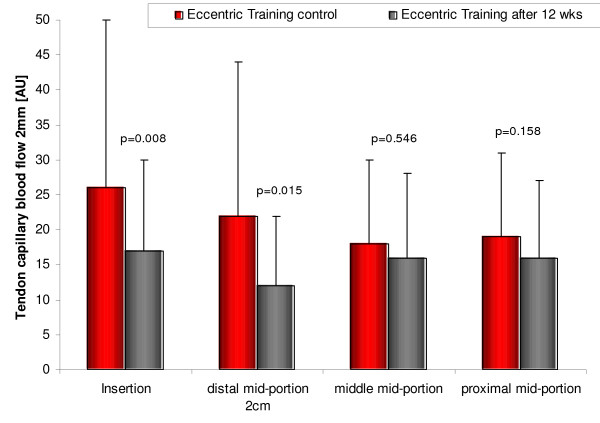
Achilles tendon capillary blood flow at 2 mm depth before (left) and after (right) 12 weeks of daily painful eccentric training in chronic Achilles tendinopathy among 59 patients with symptomatic 64 tendons.

**Figure 8:**
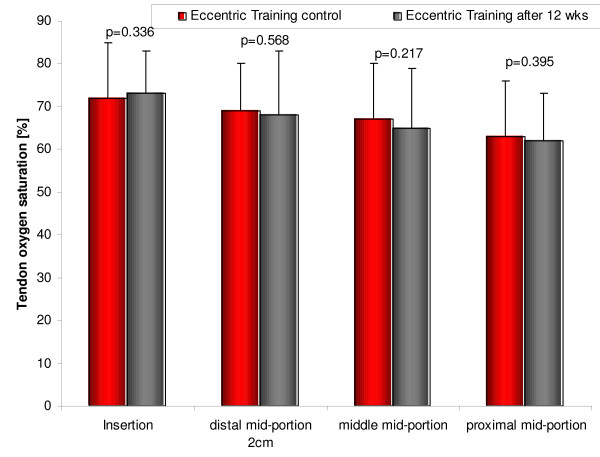
Achilles tendon oxygen saturation before (left) and after (right) 12 weeks of daily painful eccentric training in chronic Achilles tendinopathy in 59 patients with symptomatic 64 tendons.

Eccentric training is used to treat Achilles, patella, shoulder [55], and wrist tendinopathy. On a microcirculatory level, Achilles tendon capillary blood flow was significantly reduced at the insertion (by 35%, p = 0.008, figure 7) and the distal mid-portion area (by 45%, p = 0.015) at 2 mm and by 22% (p = 0.007) and 13% (p = 0.122) at 8 mm tissue depths [56]. Achilles tendon oxygen saturation was not decreased after the 12-week-eccentric-training regimen throughout the insertion to the proximal mid-portion area (insertion: 72 ± 13 vs.73 ± 10, proximal mid-portion 63 ± 13 vs.62 ± 11, both n.s., figure 8). Achilles tendon postcapillary venous filling pressures were significantly reduced at the insertion (51 ± 16 vs.41 ± 19, p = 0.001) and the distal mid-portion (36 ± 13 vs.32 ± 12, p = 0.037) at 2 mm and at the insertion at 8 mm (63 ± 19 vs.51 ± 13, p = 0.0001). No increase of tendon postcapillary venous filling pressure was noted which would be harmful.

##### Non-invasive conservative cryo/compression

Twenty-six subjects were included (32.3 ± 12 yrs, BMI 25.4 ± 5) with three ten-minute applications simultaneous cryotherapy and compression using the Aircast™ Cryo/Cuff ankle device, each followed by a 10 minute recovery period and continuous real-time assessment of parameters of Achilles tendon mid-portion microcirculation using a laser-Doppler-spectrophotometry-system (O2C, Germany) [53].

Superficial tendon oxygen saturation dropped significantly from 35.9 ± 21% to 13.5 ± 15/15.9 ± 16 and 11.1 ± 11% (p = 0.0001) during each period of cryo-compression respectively with significant increase during recovery period (55.4 ± 29/65.2 ± 26 and 65.7 ± 27%, p = 0.003) up to +83% of the baseline level. At 8 mm tendon depth, cryo-compression preserved local oxygen with -4% (p = 0.001) of the baseline level and small, but significant increased oxygen saturation of up to +13% (p = 0.0001). Relative postcapillary venous tendon filling pressures were favourably reduced to 57 ± 34%/67 ± 27 and 64 ± 38% (p = 0.0004) superficially and deep (76 ± 13%/79 ± 11 and 78 ± 18%, p = 0.0002). Superficial capillary blood flow was reduced from 48.4 ± 48 to 5 ± 7/4 ± 5 and 3 ± 4 (-94%, p = 0.0003) with increased flow during recovery periods of up to 58 ± 64/58 ± 79 and 47 ± 71 (+20%, p = 0.265). Deep flow was reduced from 197 ± 147 to 66.7 ± 64/55 ± 46 and 43 ± 39 (-78%, p = 0.0002) without increase during recovery periods.

Intermittent Cryo/Cuff™ administration of 3 × 10 min significantly decreased local Achilles tendon capillary blood flow by 90% with a subsequent small hyperaemia. Postcapillary venous filling pressures are reduced during Cryo/Cuff™ favouring venous outflow. Deep Achilles tendon oxygen supply is not impaired by Cryo/Cuff™ which is beneficial. Therefore, Cryo/Cuff™ exerts beneficial effects on the microcirculatory level of the mid-portion Achilles tendon with decreased capillary blood flow, preserved deep tendon oxygen saturation and facilitated venous capillary outflow.

##### Non-invasive conservative Achilles wrap

**Figure 9:**
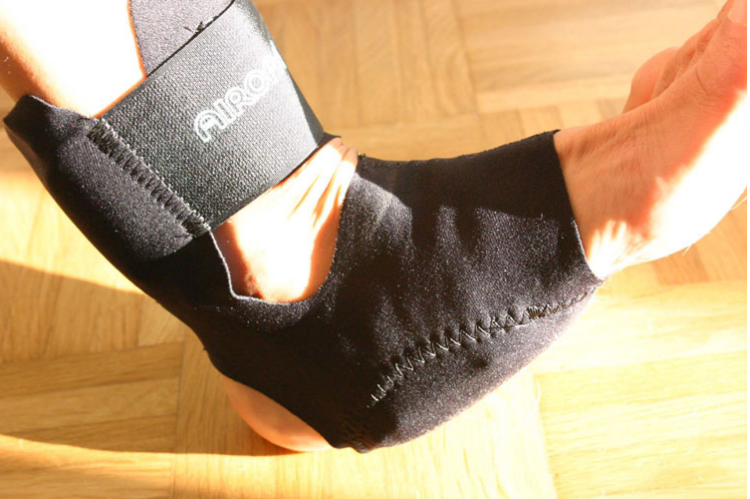
Oxygen-to-see system to determine capillary blood flow, tendon oxygen saturation and tendon postcapillary venous filling pressures non-invasively using combined Laser Doppler flowmetry and spectrophotometry.

112 subjects were recruited in a prospective randomized yet unpublished trial (figure 9). We hypothesized whether the additional use of an Achilles wrap with two interconnected air cells located under the foot arch and at the Achilles tendon, in addition to a daily eccentric training is superior than an eccentric training alone over a 12-week period concerning subjective assessment of impairment according to the Foot and ankle outcome score, pain and the microcirculatory parameters tendon capillary blood flow, tendon postcapillary venous filling pressure and tendon oxygen saturation.

Group A performed daily eccentric training over 12 weeks with additional daily Achilles wrap (AirHeel™, AIRCAST, 54 tendons of 54 patients), while group B performed the same eccentric training only (64 tendons of 59 patients). 91 patients fulfilled the 12-week-training period (81%). Tendon oxygen saturation increased significantly in group A at insertion (70 ± 11 vs.75 ± 7%, p = 0.001) and distal mid-portion (68 ± 12 vs.73 ± 9%, p = 0.006), which was significantly increased vs. group B distal mid-portion (69 ± 11 vs.68 ± 15%, p = 0.041 vs.A). Postcapillary venous filling pressures were significantly reduced in group A at 5/8 positions at two and eight mm tendon depths (up to 26%, p = 0.003), while only in 3/8 positions in group B (up to 20%, p = 0.001). Pain on VAS was 5.1 ± 2.1 vs. 3.2 ± 2.7 (A, -37.3%, p = 0.0001) vs. 5.5 ± 2.1 vs. 3.6 ± 2.4 (B, p = 0.0001, -34.6%, p = 0.486 for A vs. B).

Tendon oxygen saturation is increased and capillary venous clearance facilitated using an Achilles wrap additionally to a daily 12-week eccentric training. Achilles wrap and eccentric training increase subjective assessment of Achilles tendinopathy, however, the pain level reduction was the same in both groups with no additive effect. These results are supported by a similar recently published randomized trial with either AirHeel™ wrap or eccentric training or the combination of both [57].

##### Non-invasive topical nitroglycerin in tendinopathy

The rationale for the use of topical transdermal nitroglycerin in tendinopathy is based on the following animal studies. An increased NOS expression was demonstrated by Lin and coworkers from Sydney, Australia, at both protein and mRNA levels during Achilles tendon healing in macrophages and fibroblasts as well as in the vascular endothelial cells. All three NOS isozymes were expressed in a temporal manner in fibroblasts at the healing tendon [58].

The first published study to show an effect of 5 mg-nitroglycerine patch applied daily for three days was performed in Santander, Spain among 20 patients with supraspinatus tendinopathy with improvement on VAS from 7 ± 0.4 to 2 ± 0.3 within 48 hours [59]. The Orthopedic Research Team at St. George Hospital in Sydney has started in 2003 to publish randomized-controlled trials regarding the convincing effects of topical nitric oxide application via a patch in tennis elbow [60], mid-portion Achilles tendinopathy [61], and supraspinatus tendinopathy [62].

Recently, Paoloni and Murrel reported their three-year follow-up data in patients suffering non-insertional Achilles tendinopathy undergoing topical glyceryl trinitrate treatment over 6 months [63]. Topical glyceryl trinitrate treatment has demonstrated efficacy in treating chronic noninsertional Achilles tendinopathy, and the treatment benefits continue at 3 years. Significant differences in asymptomatic patient outcomes for the glyceryl trinitrate group continue at 3 years, and this is confirmed by the effect size estimate. This suggests that the mechanism of action of topical glyceryl trinitrate on chronic tendinopathies is more than an analgesic effect.

To date, it is unknown whether transdermal nitroglycerine affects tendon microcirculation besides the above mentioned action as a small diffusible molecule. In analogy to its long-time proven efficacy in vasodilatation of coronary arteries in coronary artery disease one could speculate that mainly the capillary blood flow is affected by NO as a vasodilatory effect. This would imply an increased, rather than a decreased capillary flow to the tendinopathic tendon, which seems, based on the current results, to be not beneficial. Furthermore, one could speculate that the vasodilatation is effective for the postcapillary venous system, which will decrease postcapillary venous filling pressures and thus, facilitating clearance of metabolic end products which is favourable. However, to date no microcirculatory data are available on this issue.

##### Non-invasive low level laser therapy

In 1998, a randomized, double-blinded, placebo-controlled study was performed in the Mayo Clinic, Rochester, MN in a sports medicine clinic [64]. 32 patients with plantar fasciitis of more than one year duration were enrolled. Low-intensity infrared laser therapy appeared safe but not beneficial regarding morning pain, pain with toe walking, tenderness to palpation, windlass test response, medication consumption, and orthotic use within one months after a for week low level laser therapy.

Recently, two randomized trials were published studying the effect of low level laser therapy with 904 nm laser on Achilles tendinopathy [65,66]. Power Doppler sonography identified peritendinous and intratendinous arterial blood flow velocity, which was used to calculate the arterial resistive index which is supposed to be a measure of vasodilatation and inflammation as (systolic peak velocity minus end diastolic velocity)/systolic peak velocity, which was possible at baseline in eight of 14 tendons. The resistive index in these eight tendons at baseline was 0.91 (95% CI 0.87 to 0.95) indicating a small degree of inflammation.

At baseline, all 14 tendons exhibited an increased peritendinous and intratendinous blood flow. After treatment, the tendinous blood flow appeared to be reduced, however, no significant differences could be found between the low level laser group and the placebo group with only 8 of 14 tendons tested. Prostaglandin E2 levels as a potential marker of inflammation were reduced following low level laser therapy. Regarding the mechanism of action, no detailed information was given.

Low level laser therapy has been shown to affect many subcellular and cellular processes, although the mechanisms have not been well defined [67]. Low level laser therapy may have physiologic effects mediated by photochemical actions at the cellular level in animal and human tissues, up-regulating cartilage proteoglycan, collagen, noncollagen protein, and DNA synthesis in the absence of histologic or biochemical evidence of enhanced matrix catabolism in animal studies [68]. However, it is important to note that LPLT does not produce significant tissue temperature changes, so any potential physiological effects appear to be nonthermal [69]. Therefore, besides effects on matrix matrix-metalloproteinases, this non-invasive technique might interfere somehow with the neovascularisation, may be decreasing the capillary flow by local thrombosis or partial destruction of the neovessels. Further studies using the detailed microcirculatory mapping might elucidate this issue.

• Invasive sclerosing/coagulation therapy focussing the area of neovascularisation

Two uncontrolled pilot studies have been published by Ohberg and Alfredson from Umea, Sweden, in which a sclerosant agent (polidocanol) was injected outside the Achilles tendon into the area of neovascularization both in mid-portion and insertional Achilles tendinopathy [70,71]. The injections were effective at reducing levels of pain, presumably as the sclerosant injection was toxic both to the neovascularization and localized sensory nerves.

A randomized-controlled trial was recently published with 32 patients with 42 tendons with chronic patellar tendinopathy enrolled from Norwegian elite basketball, handball, and volleyball divisions [72] studyng polidocanol sclerosing vs. lidocaine/epinephrine injections under colour Doppler guidance. Sclerosing with polidocanol was performed in the area of neovascularisation resulting in an improved knee function and reduced pain in the polidocanol group in contrast to the lidocaine/epinephrine group.

Two-year follow-up data have been published by Alfredson's group recently for polidocanol sclerosing therapy in mid-portion Achilles tendinopathy [73]. They concluded that treatment with sclerosing polidocanol injections in patients with chronic painful mid-portion Achilles tendinosis showed remaining good clinical results at a 2-year follow-up. Decreased tendon thickness and improved structure after treatment, might indicate a remodelling potential.

Whether tendon sclerosing technique causes local thrombosis, which would be appreciated by increased postcapillary venous filling pressures, or a local destruction of the capillary flow monitored by decreased capillary flow velocity, is currently not known. The fact that no hematoma or organized fluid is appreciated following the sclerosing technique has to be kept in mind. Interestingly, Alfredson and Öhberg reported about an increased vascularity in the early period, which is 1–3 weeks after sclerosing therapy for Achilles tendinopathy. Gradually afterwards, in between weeks 4 to 12, the neovascularisation is supposed to be fading. Therefore, the currently recommend a 6–8 week interval in between the consecutive polidocanol injections outside the tendon in order to not interfere with this process. Whether the agent injected matters is currently undetermined. There have been reports on sonographically guided intratendinous injection of hyperosmolar dextrose to treat chronic Achilles tendinopathy [74]. However, potential adverse effects such as tendon necrosis or tendon low-grade infection should be taken into account using intratendinous rather than extratendinous injections [75]. A recent randomized, controlled trial in tennis elbow tendinopathy found that colour doppler guided injections of either polidocanol or lidocaine plus epinephrine gave similar results in terms of pain reduction and voluntary grip strength [76]. Therefore, both volume, dosage and type of agent injected under colour Doppler control should be in focus for further randomized-controlled trials in the treatment of tendinopathy [77].

## Conclusion

**Figure 10:**
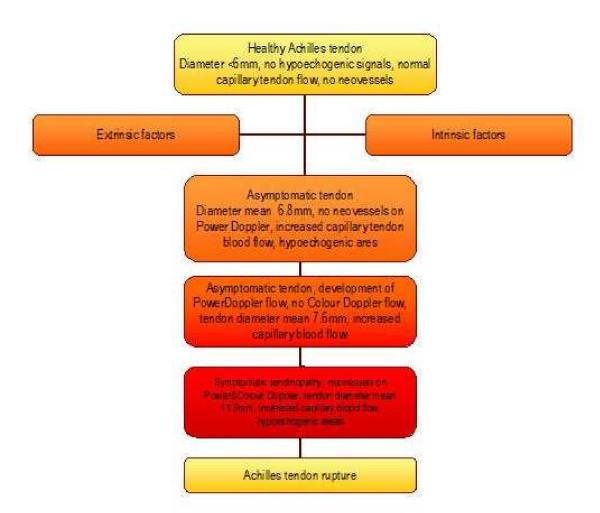
Proposed flow chart of tendon degeneration starting with the healthy Achilles tendon and extrinsic a nd/or intrinsic factors over asymptomatic states with increase in tendon diameter and detectable capillary blood flow and/or Power Doppler flow to symptomatic states and consecutive tendon rupture. Created by Knobloch with accomplishments to Richards et al. 2005, Maffuli et al. 2000, Kannus et Josza 1991.

Changes of microcirculation are evident in tendinopathy at the Achilles and the patella tendon as well as in tendionpathies of the upper extremity. Tendon capillary blood flow is increased at the point of pain correlating to neovascularisation determined by Power Doppler Sonography in Achilles tendinopathy (figure 10). Tendon oxygen saturation as well as tendon postcapillary venous filling pressures, determined non-invasively using combined Laser Doppler flowmetry and spectrophotometry, can identify therapeutic changes or adverse effects on tendon microcirculation in a real-time quantitative exact way. Eccentric training decreases pathological increased capillary tendon flow without deterioration of local tendon microcirculation in Achilles tendinopathy. Further studies have to focus on challenging therapeutic options, such as non-invasive nitroglycerine application, non-invasive laser therapy, and invasive sclerosing therapy in tendinopathy.
